# Evolutionary History of RNA Modifications at N6-Adenosine Originating from the R-M System in Eukaryotes and Prokaryotes

**DOI:** 10.3390/biology11020214

**Published:** 2022-01-28

**Authors:** Congshan Liu, Jianping Cao, Haobing Zhang, Jianhai Yin

**Affiliations:** National Institute of Parasitic Diseases, Chinese Center for Disease Control and Prevention (Chinese Center for Tropical Diseases Research), NHC Key Laboratory of Parasite and Vector Biology, WHO Collaborating Center for Tropical Diseases, National Center for International Research on Tropical Diseases, Shanghai 200025, China; liucs@nipd.chinacdc.cn (C.L.); caojp@chinacdc.cn (J.C.); zhanghaobing2@163.com (H.Z.)

**Keywords:** RNA modifications, epigenetics, m^6^A, RNA methyltransferases, demethylases, evolution

## Abstract

**Simple Summary:**

The m^6^A is the most abundant and well-studied modification of mRNA, and plays an important role in transcription and translation. It is known to be evolutionarily conserved machinery present in the last eukaryotic common ancestor (LECA). The writers and erasers responsible for adding or removing m^6^A belong to specific protein families, respectively, suggesting that these members are evolutionarily related. However, only some of these mRNA m^6^A modification-associated proteins have been studied from an evolutionary perspective, while there has been no comprehensive and systematic analysis of the distributions and evolutionary history of N6mA-associated proteins in the three kingdoms of life. In this study, we identified orthologues of all the reported N6mA-associated proteins in 88 organisms from three kingdoms of life and comprehensively reconstructed the evolutionary history of the RNA N6mA modification machinery. The results demonstrate that RNA N6mA-MTases are derived from at least two different types of prokaryotic DNA MTases (class α and β MTases). As the m^6^A reader, YTH proteins may be acquired by LECA from an individual prokaryotic YTH-domain protein that evolved from the N-terminals of an R-M system endonuclease. In addition, the origin of eukaryotic ALKBH family proteins is inferred to be driven by at least two occasions of independent HTG from the bacterial ALKB family.

**Abstract:**

Methylation at the N6-position of adenosine (N6mA) on mRNA (m^6^A) is one of the most widespread, highly selective and dynamically regulated RNA modifications and plays an important role in transcription and translation. In the present study, a comprehensive analysis of phylogenetic relationships, conserved domain sequence characteristics and protein structure comparisons were employed to explore the distribution of RNA N6mA modification (m^6^A, m^6,6^A, m^6^Am, m^6, 6^Am and m^6^t^6^A)-associated proteins (writers, readers and erasers) in three kingdoms of life and reveal the evolutionary history of these modifications. These findings further confirmed that the restriction-modification (R-M) system is the origin of DNA and RNA N6mA modifications. Among them, the existing mRNA m^6^A modification system derived from the last eukaryotic common ancestor (LECA) is the evolutionary product of elements from the last universal common ancestor (LUCA) or driven by horizontal gene transfer (HGT) from bacterial elements. The subsequent massive gene gains and losses contribute to the development of unique and diverse functions in distinct species. Particularly, RNA methyltransferases (MTases) as the writer responsible for adding N6mA marks on mRNA and ncRNAs may have evolved from class α and β prokaryotic “orphan” MTases originating from the R-M system. The reader, YTH proteins that specifically recognize the m^6^A deposit, may be acquired by LECA from an individual prokaryotic YTH-domain protein that evolved from N-terminals of an R-M system endonuclease. The eraser, which emerged from the ALKB family (ALKBH5 and FTO) in eukaryotes, may be driven by independent HTG from bacterial ALKB proteins. The evolutionary history of RNA N6mA modifications was inferred in the present study, which will deepen our understanding of these modifications in different species.

## 1. Introduction

As described by Francis Crick’s central dogma, RNA is the only direct product of DNA and is subsequently exported from the nucleus to the cytoplasm, where mRNA is translated into proteins [1,2]. RNA is also known to be a key player in transcription, translation and cellular decision-making in the form of noncoding RNAs (ncRNAs), such as rRNAs, tRNAs, microRNAs, piRNAs and long noncoding RNAs [3,4]. During these processes, gene activity, expression and function can be regulated not only by alterations in RNA sequences but also by the mechanism that is not associated with any change in the nucleic acid sequence itself, which is referred to as RNA modifications or ‘RNA epigenetics’ [5]. Chemical modifications are found in almost all types of RNA, adding additional complexity to the information carried by RNA. These modifications influence the RNA structure and its interaction with other molecules, ultimately giving rise to the diverse functions of RNA molecules, especially within complex regulatory networks, where small, subtle structural changes can bring about significant changes in cellular metabolism by affecting mRNA stability, splicing, translational efficiency and pri-microRNA processing, among other processes [6,7].

**Figure 1:**
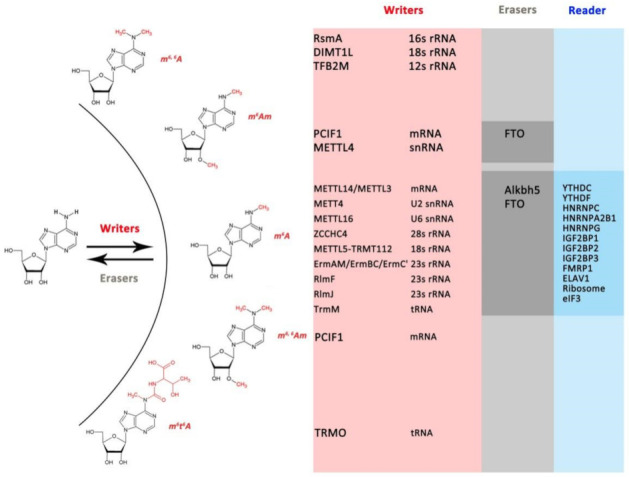
RNA methylation at N6 position. m^6^Am: N6,2′-O-dimethyladenosine; m^6^t^6^m: N6-methyl-N6-threonylcarbamoyladenosine; m^6^A: N6-methyladenosine; m^6^,^6^A: N6,N6-dimethyladenosine; m^6^,^6^Am: N6,N6,2′-O-trimethyladenosine.

RNA modifications occur across Eukaryotes, Bacteria and Achaea, indicating the conservation and importance of modifications at the RNA level [8]. Among a multitude of RNA modifications, m^6^A, one form of methylation at the N6 position of adenosine (N6mA) (Figure 1) on mRNA, was first discovered in the 1970s [9,10]. However, its functional importance and related enzymes have remained mysterious for decades until recently [8]. With a growing understanding of the functional significance of m^6^A modification in mRNA, the identification of m^6^A methyltransferase (METTL3/METLL14 complex) [11] and demethylase (FTO and ALKBH5) [12,13], and the development of next-generation sequencing (NGS), which allowed the m^6^A landscape to be delineated in the transcriptome [14,15], m^6^A has re-emerged at the forefront. It has been demonstrated that m^6^A is one of the most widespread internal modifications of messenger RNAs (mRNAs) and ncRNAs [6,16] and is highly selective and dynamically regulated in all aspects of physiological functions [17]. For instance, m^6^A play roles in protein homeostasis, proper cellular signaling and so on by affecting mRNA stability, mRNA structure, mRNA export, translation efficiency and translation [6,7,17], similar to other RNA modifications. Moreover, mutations in m^6^A modification enzymes have been linked to human diseases, including cancer, cardiovascular diseases, metabolic diseases and neurological disorders, highlighting their importance under both physiological and pathological conditions [18]. Recently, studies on other types of N6mA, such as m^6,6^A, m^6^Am, m^6,6^Am and m^6^t^6^A (Figure 1), have revealed their key roles in regulating mRNA stability, pre-mRNA splicing and the stress response [19,20,21], adding the complexity and diverse function of N6mA RNA modifications.

The pattern of m^6^A in mRNA relies on the complex interactions among three distinct classes of protein factors: “writers”, which are m^6^A methyltransferases (MTases); “erasers”, which are m^6^A-demethylating enzymes that mediate dynamic modifications; and “readers”, which are m^6^A-binding proteins that recognize and regulate modifications, contributing to their cellular effects. The best-studied “writer” executes the deposition of m^6^A modifications via a multicomponent complex (MTC) composed of a METTL3-METTL14 heterodimer and regulatory factors, including WTAP, VIRMA, ZC3H13, RBM15 and HAKAI, by catalyzing the addition of a methyl group to adenosine in the context of a conserved consensus sequence (RRACH) [17,22]. In this complex, WTAP stabilizes the interaction between the two METTL proteins, while the other regulatory factors have been proposed to function in guiding methylation to targeted sites [22]. To date, only two demethylases, FTO and ALKBH5, have been known to remove mRNA m^6^A modifications [12,13]. Moreover, YTHs, HNRNPs, IGF2BPs, eIF3, FMRP1, ELAV1 and ribosomes affect the fate of RNAs by acting as m^6^A “readers” [17,23]. Together, these elements constitute the core mRNA m^6^A modification components, regulating the functions of this modification. Recently, other MTases, such as METTL16, ZCCHC4 and METTL5, have been identified as m^6^A MTases that can catalyze the deposition of m^6^A marks on ncRNAs [2,24]. In addition, writers and erasers of other N6mA have been identified [20,21,25,26,27,28,29] (Figure 1), but no readers have yet been found.

The mRNA m^6^A modification is known to be an evolutionarily conserved machinery present in the last eukaryotic common ancestor (LECA), according to comparative analyzes of m^6^A-associated proteins and their modification sites across closely related species [8,18,30] and very distant organisms [2]. Furthermore, the writers and erasers that regulate N6mA modifications belong to specific protein families, suggesting that these members are evolutionarily related. However, only some of them have been studied evolutionarily [31,32,33,34,35,36], and there has been no comprehensive and systematic analysis of the distributions and evolutionary history of N6mA-associated proteins in the three kingdoms of life. In this study, we identified homologues of N6mA-associated proteins (also referring to the components of N6mA modifications) in 88 organisms from three kingdoms of life and comprehensively reconstructed the evolutionary history of the RNA N6mA modification machinery, including writers (MTases), erasers (ALKHB family) and readers (YTH-domain-containing protein).

## 2. Materials and Methods

### 2.1. Sequence Similarity Searches

Candidate homologues of known m^6^A, m^6,6^A, m^6^Am, m^6, 6^Am and m^6^t^6^A writers, readers and erasers (Figure 1, Table S1) were identified by scanning a panel of 88 taxa (Table S2, including 17 Bacteria, 6 Archaea, 7 Discoba, 2 Metamonada, 14 SAR, 6 Archaeplastida and 36 Amorphea). The list of 47 reported and well-studied prokaryote and human protein sequences involved in these RNA modifications were used as an initial set (accession numbers for initial queries are provided in Table S1). Homologues were identified by using web-based PSI-BLASTP searches among NCBI non-redundant protein sequences (https://blast.ncbi.nlm.nih.gov/Blast.cgi, accessed on 10 January 2021), with an E-value cut-off < 0.05. For *Euglena gracilis*, which lacks a high-quality genome in NCBI, the sequences were extracted from the proteome database (http://proteomecentral.proteomexchange.org/cgi/GetDataset?ID=PXD009998, accessed on 25 January 2021) with the basic local alignment search tool (BLAST 2.2.25) (https://blast.ncbi.nlm.nih.gov/Blast.cgi, accessed on 10 January 2021). All hits (E-value cut-off < 0.05) that were obtained for the same species were pooled, and redundant sequences were deleted using the CD-HIT suite [37] with a cut-off of 0.98. To determine the true sequences in the BLAST hits, only the sequences containing the domains as the initial queries (Table S3) were retained. The domain research was carried out by Batch CD-Search at NCBI (https://www.ncbi.nlm.nih.gov/Structure/cdd/wrpsb.cgi, accessed on 10 January 2021). There was one exception, ZC3H13, which lacked the reported domain in some species. Only the top hits were used for the phylogenetic analysis. The amino acid residues were visualized with Jalview Version 2 [38].

### 2.2. Alignment Generation and Phylogenetic Analyzes

Sequences were aligned using the web-based COBALT tool based on the conserved domain and local sequence similarity [39] with default parameters. To infer the evolutionary history of some protein families, such as the AdoMet_MTases superfamily, ALKBH family, YTHs, HNRNPs and IGF2BPs, the homologous sequences of all the species were pooled together before aligning the sequences. PCIF1 is not included in the phylogenetic tree of AdoMet_MTases because of its long branch attraction. These alignments were manually trimmed to retain only the columns with at least 30% letters in a column that were not gap characters and at least 10% of the pairs of letters in an alignment column that were conserved (Table S4). The alignments used for phylogenetic analyzes can be found in Supplementary File S1.

The online IQ-TREE platform [40], an algorithm to infer phylogenetic relationships by maximum likelihood, was used to find the best fitting model (Table S4) and build phylogenetic trees. The tree branches were tested with ultrafast bootstrapping (1000) and SH-like approximate likelihood ratio tests (SH-aLRT, 1000 replicates). The final trees were visualized with TreeGraph2 [41]. Gene gains and losses were inferred based on phylogenetic trees and conserved domains (sequence architecture). Using the core domains, the specific human tree of N6mA modifications related to AdoMet_MTases and the ALKBH family was also generated to facilitate visualization of the different families.

Orthologues, paralogues, xenologues and gene losses were further inferred according to the phylogenetic topologies. Homologous sequences that have diverged due to speciation events were identified as orthologies. The duplicated genes are searched through the identification of paralogous relationships. After comparing with the putative history of the species, xenologues were identified as genes in one species derived from a distantly related species through HGT. To minimize the genome quality effects, only the lineage-specific losses were defined as gene losses.

In order to ensure these gene losses are contributed by evolution rather than limitation of methods and genome quality, this study make the following endeavors: (1) In order to include as much sequences of studied species as possible, NCBI’s non-redundant protein sequences, including all non-redundant GenBank CDS translations + PDB + SwissProt + PIR + PRF excluding environmental samples from WGS projects search sequences were used in the sequences searching; (2) All sequences with E-value > 0.05 rather than the top hits were included when we searched for homologues, and then a series of screenings and phylogenetic relationship analysis were used to ensure the integrity of the included sequences and the accuracy of classification in considered species; (3) The results have been validated through model organisms, including human, *D. melanogaster*, *C. elegans* and *S. cerevisiae*, which has been reported as the m^6^A modification-associated elements and sequences; (4) Only the lineage-specific losses were identified as gene losses, so as to exclude sequence losses due to the genome quality; (5) NCBI and Ensemble are the two completed and qualified genome database, and also the main database used for orthology identification, such as OrthoMAM (https://orthomam.mbb.cnrs.fr/, accessed on 25 January 2021) and OrthoDB (https://www.orthodb.org/orthodb_userguide.html, accessed on 25 January 2021), so we searched the homologues of m^6^A associated protein in prokaryotes with Ensemble database (Table S5).

### 2.3. Structural Inferion and Similarity Comparison

Data for experimentally determined structures (Table S1) were retrieved from the PDB database (https://www.rcsb.org, accessed on 25 January 2021). Data for other structures without released crystal structures were extracted from the SIWSS-MODEL Repository database [42] (Table S1). Detailed information is summarized in Supplementary Table S1. These structures were visualized, and the similarity of protein structures, represented as the root mean square deviation (RMSD), was calculated using Open-Source PyMoL [43].

### 2.4. Structure-Based Alignments

For sequences with diverse amino acid sequences but similar topologies, PROMALS3D was used to accurately build multiple alignments based on the released 3D structure [44].

## 3. Results

### 3.1. The Distribution of N6mA Modification Components in Prokaryotes and Eukaryotes

#### 3.1.1. mRNA m^6^A Modification Components in Prokaryotes and Eukaryotes

**Figure 2:**
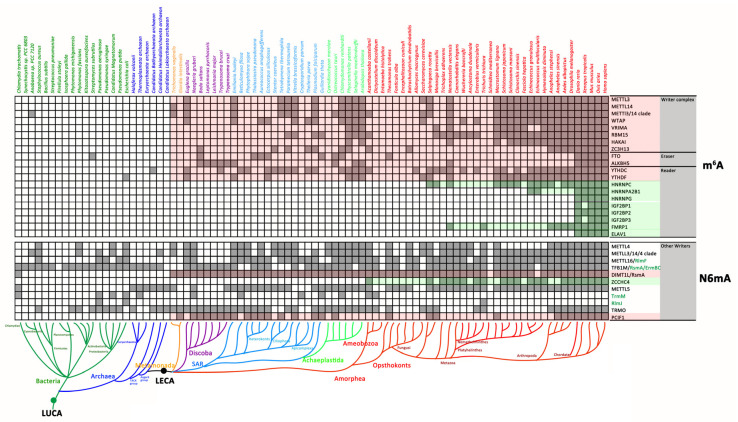
Distribution of RNA N6mA modification components in prokaryotes and eukaryotes. The landscape map was delineated based on Figures S1–S14. The red background highlights the gene gains before LECA; the green background highlights the gene gains in Amorphea.

The homologues of mRNA m^6^A modification-associated proteins (Figure 1) are found in only eukaryotic genomes rather than prokaryotes (Figure 2), suggesting that the mRNA m^6^A modification system originated from LECA. As the core components, the writer complexes METTL3/METTL14, WTAP and RMB15, readers YTHDCs and YTHDFs, and erasers FTO and ALKBH5 are conserved in the major phylum of eukaryotes. However, some mRNA m^6^A modification-associated proteins are lineage-specific. For instance, HNRNPC and FMBP1 were identified in organisms that emerged evolutionarily after *Salpingoeca rosetta* and *Nematostella vectensis*, which are the closest single-celled relatives or progenitors of Metazoa, and were retained in the majority of Metazoa during subsequent evolution. HNRNPA2B1, HNRNPG, IGF2BPs and ELAV1 could be identified only in vertebrates, suggesting that they might be gene gains in the common ancestor of vertebrates. VIRMA and HAKAI were highly divergent between plants and animals and were not present in lower eukaryotes (Figure 2). Although most mRNA m^6^A modification-associated proteins were absent in prokaryotes, only with some sporadic m^6^A modification components identified in Bacteria or Archaea, such as METTL3/METTL14 in *Anabaena PCC 7122*, FTO in *Streptomyces subrutilus*, YTHDC in *Candidatus Bathyarchaeota archaeon* and YTHDF in *Escherichia coli*. Hence, the spread of these genes might be mediated by horizontal gene transfer (HGT) between eukaryotes and prokaryotes (Figure 2, Figures S1, S9 and S16).

The METTL3-METTL14 heterodimer was lost in some species’ lineages, such as kinetoplastida, heterokonts, fungus and nemathelminthes (Figure 2). Interestingly, the gene loss patterns of METTL3/METTL14, WTAP and the YTH family (especially YTHDC) were highly consistent (Figure 2), indicating that they coevolved as the core elements of mRNA m^6^A modification. In addition, both FTO and ALKBH5 were lost in most Amorphea (except for vertebrates) as well as in Metamonada, early Discoba and some SAR taxa (Figure 2).

#### 3.1.2. The Distribution of Other N6mA-MTases in Prokaryotes and Eukaryotes

The majority of other N6mA-MTases, such as METTL4, METTL16, METTL5, TFB1M, TrmM and TRMO, might have originated in the early stages of evolution prior to the last universal common ancestor (LUCA), while DIMT1L/RsmA and PCIF1 were only found in eukaryotes, which were traceable to LECA (Figure 2). In addition, the distribution of some N6mA-MTases was lineage-specific; for instance, ZCCHC4 was present in only Amorphea, TrmM was lost in Opsthokonts, and RlmJ was a bacteria-specific m^6^A methylase (Figure 2). Furthermore, unlike METTL3-METLL14, these N6mA-MTases are mainly modified ncRNAs rather than mRNAs, suggesting that ncRNA modifications are conserved machinery with prokaryotic origins and are retained in eukaryotes.

### 3.2. The Evolutionary History of the RNA N6mA Modification Machinery

#### 3.2.1. The Writer: RNA N6mA-MTases

**Figure 3:**
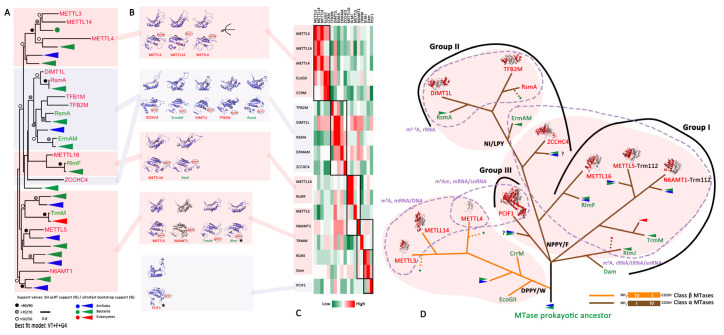
Evolution and characteristics of RNA N6mA methyltransferases. (**A**) The phylogenetic tree of RNA N6mA-MTases (details in Figure 1). (**B**) The structure of the catalytic domain of MTases. The conserved four-residue consensus is labeled in red. RFMs use AdoMet as the methyl group donor and show a conserved catalytic mechanism: a four-residue consensus (D/N/S/H-PP-W/F/Y) is generated in motif IV (methylation catalysis motif), where the bulky hydrophobic side chain (W/F/Y) stacks against the target base with π-π interactions, while the polar group of the first residue (D/N/S/H) abstracts a proton from the target NH_2_ group. (**C**) Structural comparison of MTD in MTases. A heatmap was drawn based on the root mean square deviation (RMSD). (**D**) The hypothesis of the evolutionary history of N6mA MTases. RFMs can be categorized into Class α (DNA MTase Dam), β (DNA MTase CrrM) and γ according to their circular permutation in the order of motifs. Class α Dam, brown branch; Class β EcoGII, orange branch. There are three groups of Class α N6A MTases according to their structural similarity and four conserved residues. MTases in the same background retain similar catalytic motifs.

AdoMet-dependent MTases are divided into five structurally distinct classes: I (Rossmann-Fold MTases, RFM), II, III, IV (SPOUT) and V [45]. Among them, only RFM and SPOUT MTases can methylate RNA [32,33]. TrmO, with a unique single-sheeted β-barrel structure close to that of *E. coli* yaeB, was identified to add m^6^t^6^A to tRNA (Thr) [46], while other reported N6mA-MTases exclusively belong to the RFM class [31,47,48] (Figure 3). In this study, the conserved N6mA-MTases from 88 taxa were clustered into different clades in the phylogenetic tree (Figure S1). We retained the well-studied eukaryotic and bacterial RNA N6mA-MTases in a simplified tree to easily clarify their evolutionary relationships (Figure 3A). Evolutionary analysis of N6mA MTases suggested that there was a common ancestor prior to LUCA for these RNA N6mA-MTases (except TrmO). Subsequently, they were independently acquired by LECA from separate prokaryotic ancestors (except ZCCHC4 and PCIF1), which might be products of HGT (Figure 3A).

Inspired by the consensus that RNA MTase and DNA MTases came from a common ancestor in the restriction-modification (R-M) system [31,34,35,49], bacterial “orphan” MTases (Class α DNA MTase Dam, Class β DNA MTase CrrM and EcoGII) were recruited in our study for the analysis of the evolutionary relationship of RNA N6mA-MTases based on their protein structures (Figure 3). Unlike MTases in the bacterial and archaeal R-M system that pair with a restriction endonuclease for self-non-self discrimination, “orphan” MTases are involved in chromosome replication, DNA repair and epigenetic gene regulation [31,50,51]. Furthermore, although both Dam and CrrM are RFMs and act on DNA N6mA, their methylation mechanisms are quite different, indicating that they evolved independently [31], which is consistent with our finding that RNA N6mA-MTases originated from at least two types of DNA MTases, that is, Class β and Class α DNA MTases (Figure 3).

METTL3, METTL14 and METTL4, as the members of MT-A70 (Figure 3A), were inferred to originate from precursors of the DNA MTases CcrM and EcoGll for three reasons. First, all of these RNA and DNA MTases belong to class β MTases that can generate N6mA on double-stranded DNA (dsDNA), single-stranded DNA (ssDNA) or mRNA [31,52,53,54]. Second, there were more structural similarities of the methylation transferase domains (MTDs) between human METTL3/METTL14/METTL4 and bacterial CrrM/EcoGII than to other RNA N6mA-MTases (Figure 3C). Finally, there was a consistency of the motif order (IV-I) and the DPPY/W motif in their catalytic domain (Figure 3B,D). In addition, unlike METLL3-METTL14, which prefers single-stranded nucleic acids, another MT-A70 member, METLL4, is predicted to be the primary N6mA DNA MTase [55,56]. Hence, active METTL3 might represent by the gene duplication of METTL4 and enable a functional shift towards RNA methylation, followed by the emergence of inactive METTL14 as its partner.

Another group of Class α MTase (motif order is I-IV) [31,33,57], RNA N6mA-MTases, mostly containing N6mA modifications in the ncRNA, might have evolved from a precursor of DNA MTase Dam (Figure 3D). Although the EPPV motif of *E. coli* Dam differed from the NPPY motif *of E. coli* RlmJ, the root mean square deviation (RMSD) of their three-dimensional structures was 2.96 Å (Figure 3C, Table S6), indicating that these two proteins had close evolutionary relationships compared to other RNA MTases. We divided known RNA N6mA-MTases into three evolutionary groups according to their structure and functions (Figure 3D). Group I included RlmJ, TrmM, METTL5, N6AMT1, RlmF and METTL16, which contributed to m^6^A modification on ncRNAs and showed the consistent catalytic motifs NPPF/Y. Among them, some MTases have established special catalytic mechanisms and functions. For instance, METTL5 relies on Trm112 for stabilization [58], and N6AMT1/HemK-Trm112 is a protein MTase rather than a nucleic acid enzyme [59,60]. In Group II, ZCCHC4, ErmAM, RsmA, DIMT1L and TFB2M had high structural similarities (Figure 3B,C). ErmAM was thought to be the ancestor of RsmA, DIMT1L and TFB2M, all of which had consistent NI/LPY catalytic motifs. PCIF1 was the only member of Group III (Figure 3D) but was not included in the phylogenetic tree because of its long branch attraction (Figure 3A). Despite an absence of homologues in prokaryotic organisms, the NPPF motif and unique function of PCIF1 (catalyzing m^6^Am and m^6,6^Am modifications of mRNA) [25,61] indicate that it may have been acquired by eukaryotes from prokaryotes prior to their radiation from LECA.

#### 3.2.2. The Eraser: ALKBH Family Demethylases

**Figure 4:**
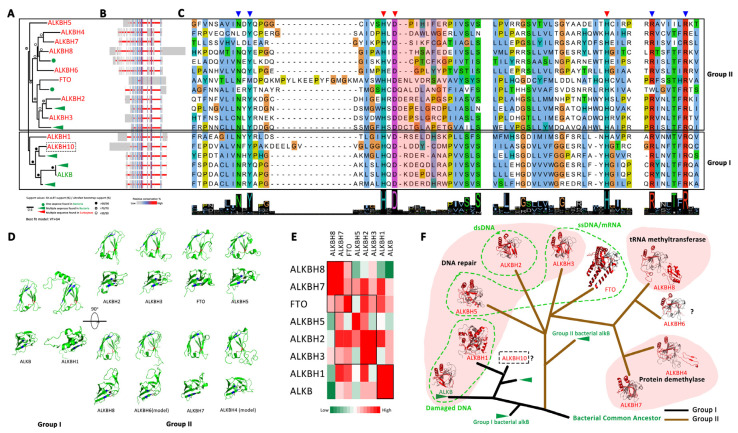
Evolution and characteristics of the ALKBH family. (**A**) The phylogenetic tree of the ALKBH family (details in Figure S9). (**B**) Alignments of the ALKBH family, ALKBH5 (NP_060228.3), ALKBH4 (NP_060091.1), ALKBH7 (NP_115682.1), ALKBH8 (NP_620130.2), *Pseudomonas syringae* KPW66023.1, ALKBH6 (NP_116267.3), FTO (NP_001073901.1), *Streptomyces subrutilus* WP_069918251.1, ALKBH2 (NP_001138846.1), *P. syringae* WP_110682285.1, ALKBH3 (NP_631917.1), *P. aeruginosa* WP_021219048.1, ALKBH1 (NP_006011.2) *Physcomitrella patens* XP_024396199.1, *Kitasatospora aureofaciens* WP_051826438.1, *P. aeruginosa* MXH34269.1, ALKB (NP_416716.1), *P. syringae* WP_191998247.1. (**C**) The amino acid in the catalytic domain. The conserved Fe^2+^-binding site residues that are marked by a red triangle are HXD-H, and the α-KG-binding site residues (N-Y-R-R) are marked by blue triangles. The red background indicates the active-site region that is critical for substrate recognition and demethylation specificity. (**D**) The structure of DBSH. HXD-H is labeled in red, and N-Y-R-R is labeled in blue. (**E**) Structural comparisons of DBSH in the ALKBH family. A heatmap was drawn based on RMSD. (**F**) The hypothesis concerning the evolutionary history of the ALKBH family, which is derived from the bacterial DNA repair enzyme ALKB but independently originates from different bacterial ALKB ancestors (enzymes with black branches originating from Group I bacterial ALKB, brown branches originating from Group II bacterial ALKB). ALKBHs in the same background show a similar catalytic function.

ALKB homologues (ALKBHs) are a family of specific demethylases that depend on Fe^2+^ and α-ketoglutarate (α-KG) to catalyze demethylation on different substrates, including dsDNA, ssDNA, mRNA, tRNA and proteins [36]. Their catalytic domain contains a conserved double-stranded β-helix domain (DSBH) known as “jelly roll” fold, which forms the metal-(HXD-H) and cofactor-binding (N-Y-R-R) pockets (Figure 4C,D). The sequence characteristics, structures and functions of the ALKB family have been well described [36,62,63,64]. Here, their evolutionary relationships are carried out in a comprehensive way by integrating their sequences and structural characteristics of the active DBSH domain in the present study.

The phylogenetic analysis confirmed that the eukaryotic ALKBH family was derived from the bacterial DNA repair enzyme ALKB, and no ALKB homologues were found in Archaea [36,65] (Figure 4, Figures S9 and S15). Importantly, we provide new insight that the eukaryotic ALKBH family evolved from different bacterial ALKB enzymes. That is, ALKBH1 and ALKBH10 originated from one bacterial ALKB group (Group I, Figure 4), while other ALKBHs originated from another bacterial ALKB group (Group II, Figure 4). In addition, we identified ALKBH10 as a novel eukaryotic ALKBH protein that was lost in metazoans during evolution (Figure S15). Considering that ALKBH10 is evolutionarily more closely related to *E. coli* ALKB than ALKBH1 (Figure 4C), we inferred that ALKBH1 arose from the gene duplication of ALKBH10 during eukaryogenesis and appeared before LECA. Therefore, the newly discovered ALKBH10 retained in lower eukaryotes might play a similar role in mediating DNA repair as human ALKBH1 and *E. coli* ALKB.

The ALKBHs predicted to evolve from Group II bacterial ALKB (brown branch in Figure 4D) were clustered into one clade in phylogenetic trees (Figure 4A). Among them, ALKBH2/ALKBH3 might emerge at earlier evolutionary stages, while others were derived via the gene duplication of ALKBH2 or ALKBH3 (Figure 4A, Figures S2 and S15). These ALKBHs underwent functional differentiation to obtain certain functions, such as the direct reversal of alkylation damage in DNA (ALKBH2 and ALKBH3), regulation of epigenetic or epitranscriptomic nucleic acid methylation (ALKBH5 and FTO) [12,13], posttranslational modifications by acetylating proteins (ALKBH4 and ALKBH7) [62,66,67] or acting as a hydroxylating enzyme on tRNA rather than as a demethylase (ALKBH8) [68].

#### 3.2.3. The Reader: YTH Family

The YTH domain, consisting of four α-helices and six β-strands, serves as the module for recognizing m^6^A in a methylation-dependent manner [69,70]. Three YTHDF and two YTHDC proteins have been identified in the human genome to recognize m^6^A markers rather than other RNA modifications [70]. These proteins were previously found only in eukaryotes [71,72], but their prokaryotic origin was recently indicated by two striking similar structures: the McrB-N domain (McrB, a methylation-dependent endonuclease in the R-M system) from the archaeon *Thermococcus gammatolerans* and MJECL36 from *Methanocaldococcus jannaschii* [73,74,75].

**Figure 5:**
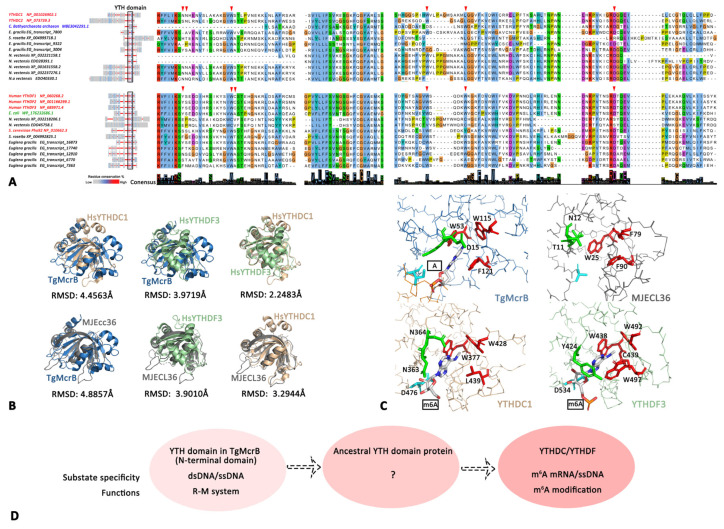
Evolution and characteristics of YTH domains. (**A**) Protein sequences of YTHDC and YTHDF in humans, prokaryotes and basal eukaryotes. The residues for recognition are marked by a red triangle. (**B**) The structural similarity of human YTHDC1 (6zcn_A), YTHDF3 (6zot_B), TgMcrB (6p0g_A) and MJECL36 (2p5d_A). (**C**) The m6A recognition aromatic cages of YTHDC1 and YTHDF3 and the corresponding amino acids in TgMcrB and MJECL36. (**D**) Hypothesis of evolution of the YTH domain containing proteins.

In the present study, YTHDF and YTHDC were found in the major phylum of eukaryotes and sporadic prokaryotes (Figure 2). The sequences of YTHDF and YTHDC in humans, basal eukaryotes and prokaryotes were well conserved (Figure 5A), while there were low amino acid sequence identities between their YTH domain and McrB-N domain or MJECL36 (Figure S16). However, the three hydrophobic amino acids forming an aromatic cage to accommodate nucleotides [73,74,75] were highly conserved among eukaryotic YTH, McrB-N domain and MJECL36 (Figure 5C, Figure S16), indicating their ancestral function of recognizing nucleotides. Furthermore, the topological structural similarities between TgMcrB and human YTH proteins (RMSD = 4.45 Å and 3.97 Å, Figure 5B) and between MJECL36 and human YTH proteins (RMSD = 3.90 Å and 3.29 Å, Figure 5B) indicated that they might have evolved from a common ancestor. In addition, the McrB-N domain can facilitate TgMcrB binding to dsDNA/ssDNA rather than RNA substrates [73], while human YTH proteins target ssDNA and mRNA. The functional transition from recognizing dsDNA to single-stranded nucleotides adds further evidence of the evolutionary origins of the m^6^A mRNA system.

As the N-terminus of the R-M system endonuclease, TgMcrB might be one of the prokaryotic origins of the eukaryotic YTH domain, and it may have subsequently evolved to individual YTH domain-containing proteins such as MJECL36. After a long period of evolution, these YTH domain-containing proteins were acquired by LECA with adaptive functions for RNA recognition (Figure 5D).

## 4. Discussion

Phylogenetic trees are widely used to infer the evolutionary relationships of species and genes. For instance, many cases of bona fide functional inter-kingdom HGT events have been documented, validating the movement of genetic information between distant species. Among the approaches to identifying HGT, phylogenetic methods are the most general and potentially most sensitive methods [76]. These methods, as we used in this study, reconstruct the history of a family of homologous genes (a gene tree, Figures S1–S16), and compare it to a putative history of the species in which the genes are found (the species tree, Figure 2) [76,77]. Homologues are genes that share a common ancestry and are divided between orthologues (derived by speciation) and paralogues (derived by duplication). Based on this definition, the search for duplicated genes can be done through the identification of paralogue relationships [78,79,80,81]. However, the diversity of genetic information caused by evolution has introduced difficulties into analyzes, especially for genes with a relatively distant evolutionary relationship. Fortunately, the released protein structures make it possible to build evolutionary relationships of functions and sequence distinct proteins according to the topological similarity of structures. In this study, phylogenetic trees and protein structures were integrated to reveal the distribution of N6mA-associated components in three kingdoms of life, predict possible modifications and functions in different species and explore the evolutionary history of mRNA m^6^A modification, providing a basis and motivation for in-depth studies of these modifications in distinctive species.

### 4.1. Eukaryotic mRNA m^6^A Modification Components Originating from LECA

**Figure 6:**
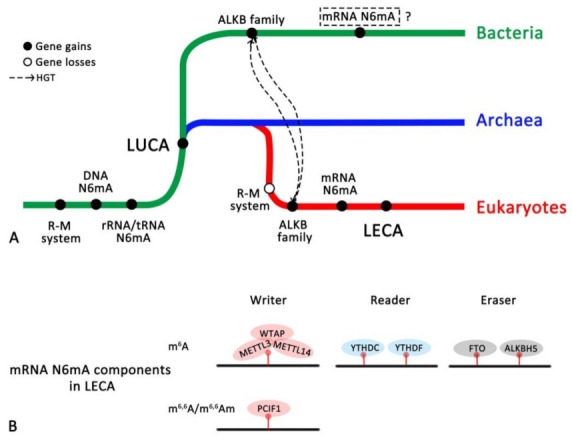
The hypothesis of the evolution of N6mA modifications. (**A**) Hypothesis of the evolution of the N6mA modification system. (**B**) Ancestral mRNA N6mA components in LECA.

The homologues of mRNA m^6^A modification-associated proteins are found in only eukaryotic genomes rather than prokaryotes (Figure 2), adding evidence to the hypothesis that the mRNA m^6^A modification system originated from LECA. The distribution of m^6^A modification core elements in distinct species suggests that the ancestral m^6^A machinery in LECA is a form composed of “ancient writer complex”, with one pair of METTL3/METTL14, one auxiliary WTAP and RBM15, ZC3H13 protein each; “ancient reader” with one YTHDC and one YTHDF; and “ancient eraser” with one FTO and one ALKBH5 (Figure 6B), which is a more complicated complex than indicated in a previously reported study [2]. This complex evolved to the extant complex in different species by recruiting additional proteins to perform distinct biological functions in heterogeneous and changing cellular environments. In parallel, gene expansion has led to the formation of lineage-specific characteristics, such as HNRNPC and FMBP1 in Metazoa, and HNRNPA2B1, HNRNPG, IGF2BPs, ELAV1 in vertebrates. VIRMA and HAKAI are highly divergent between plants and animals and are not present in lower eukaryotes. The emergence of these two proteins could represent a case of convergent evolution, similar to the occurrence of the origin of parental genomic imprinting [2].

### 4.2. The Concerto of Gene Losses and Gains Involved in the mRNA m^6^A Modification Machinery

Although a great deal of attention has been given to the mechanisms of evolution via gene duplication, gene loss is a crucial process [82]. Gene losses were also found in the evolution of mRNA m^6^A modification machinery, such as the absence of METTL3-METTL14 in some species lineages. One possible explanation for this phenomenon is the potential presence of substitute enzymes for adding m^6^A to mRNA because auxiliary components in the “writer complex” were found in some species that lost the METTL3-METTL14 heterodimer. Another possibility is that these taxa have completely lost the mRNA m^6^A modification machinery, while the remaining auxiliary components play roles in processes other than m^6^A RNA modification. For the first and substitute hypothesis, METTL4, as a member of the MT-A70-like protein family, including METTL3 and METTL14 (Figure 2), is a potential substitute enzyme. Unlike METTL3 and METLL14, METTL4 is conserved in three kingdoms of life, especially in eukaryotes lacking METTL14/METTL3 proteins (Figure 2, lower panel). In addition, METTL4 can modify N6mA in DNA or snRNA [20,55,83,84], adding to its ability to functionally compensate for the absence of METTL3 and METTL14. Kinetoplastida lost all three enzymes, but m^6^A mRNA modification has been identified in *T. brucei* [85], also raising the possibility that other N6mA MTases might functionally compensate for the absence of the METTL3/METTL14 dyad in these species. Hence, in view of evolution, as the paralogues of METTL4, METTL3 and METTL14 might originate from the duplication of METTL4 before LECA, and thereby achieve functional expansion and diversity. In some species, the loss of METTL3/14 and the retention of METTL4 may be the result of their common ancestor’s adaptation to the environment. However, for some species, the varieties of enzymes for RNA modification are needed to maintain their functional diversity, which was supported by the reported distinct function of these MTases [20,55,83,84]. Therefore, it is inferred that the genes’ gains and loss of METTL3/14 may be the result of adaptive evolution.

Both FTO and ALKBH5 may be derived from LECAs, but their absence in early-branching eukaryotes raises the following questions that need to be answered: are there other m^6^A demethylases, and is m^6^A a dynamic modification in these species? These two enzymes belong to the ALKB family [86]. The conserved catalytic domain in this protein family makes other members potential substitutes for FTO/ALKBH5 (Figure S15), but no clear evidence has been obtained thus far.

Above all, the evolution of bacterial, archaeal and eukaryotic genomes is a dynamic process that involves extensive gains and losses of gene events [82,87]. In particular, gene duplication is a fundamental process in the evolution of species and plays a major role in the origination of novel gene functions [88]. Gene losses have often been associated with the loss of redundant gene duplicates driven by evolutionary forces but have recently been suggested to be beneficial for organisms, as they can be a pervasive source of genetic variation that gives rise to adaptive phenotypic diversity [82,89]. In the present study, “writer complex”, “erasers” and some “readers” have undergone specific gene losses during evolution, while some elements in “writer complex” and other “readers” were generated by gene gains. Consequently, gene gains and losses form specific combinations of m^6^A components to provide functional diversity in distinct species by finely tuning m^6^A modifications on mRNA. Hence, during the evolution, the mRNA m^6^A system becomes more complex or simpler in some species, for instance, Chordate contains the most complex mRNA m^6^A system by gene and function expansion, while Discoba, SAR and Fungusi lost many m^6^A modification-related genes.

### 4.3. The Evolutionary History of the RNA N6mA Modification Machinery

The R-M system is the most widespread prokaryotic biological conflict system facilitating both discrimination of cellular self DNA from invasive non-self DNA and destruction of the latter [35]. The similar function and topological structure of MTase and YTH domain-containing proteins in prokaryotes and eukaryotes indicate that the R-M system in both Bacteria and Achaea is the ancestor of N6mA modification on DNA and RNA for three kingdoms of life (Figure 6A). N6mA modification of DNA is carried out by “orphan” MTases to mark and regulate replication origins of genomic and plasmid replicons, DNA repair and epigenetic gene regulation [31,35,50,51]. Subsequently, ncRNAs such as rRNA and tRNA make use of this mechanism to regulate translation. It is becoming apparent that before the split of bacteria and archaea, N6mA modifications on DNA, rRNA and tRNA, together with the R-M system, were retained in LUCA.

In the evolutionary history of eukaryotes, the emergence of mRNA N6mA components in LECA (Figure 6B) is the evolutionary product from LUCA (MTase and YTH domains) or driven by HGT from bacterial elements (ALKB family). Concomitantly, the physiological roles of mRNA N6mA were massively extended, which resulted in increases in mRNA N6mA modification-related genes in eukaryotic genomes and finally contributed to the complexity of the species. Furthermore, eukaryotes lost the R-M system in prokaryotes and retained N6mA modifications on rRNA and tRNA (Figure 6A). Although DNA methylation modification is likely coordinated with the recognition of specific histone methylation marks in lower eukaryotes [35], its existence and function are debatable issues regarding mammalian DNA [31,74].

Until now, RNA modifications in Archaea have remained mysterious, and mRNA N6mA modifications have been found in only eukaryotes and bacteria, but with quite different mechanisms because: (1) bacterial m^6^A motifs (GCCAUs) are distinct from those of mammals (RRACHs); (2) the enzymes responsible for adding m^6^A to bacterial mRNA have not been found [90]; and (3) no bacterial homologues of components associated with the mRNA m^6^A machinery were confirmed in our study.

## 5. Conclusions

The comprehensive evolutionary history of RNA N6mA modifications is delineated in the present study. The distribution of known RNA (mRNA, ncRNAs) N6mA (m^6^A, m^6,6^A, m^6^Am, m^6, 6^Am and m^6^t^6^A) components in species from the three kingdoms of life indicates the possible RNA N6mA modifications involved in the specific biological function of distinct species (Figures S1–S14). Moreover, the hypothesis that RNA N6mA-MTases are derived from at least two different types of prokaryotic DNA MTases (class α and β MTases) was further confirmed. As the m^6^A reader, YTH proteins can specifically recognize the m^6^A tag and may be acquired by LECA from an individual prokaryotic YTH-domain protein that evolved from N-terminals of an R-M system endonuclease. In addition, the hypothesis that the origin of eukaryotic ALKBH family proteins is driven by at least two occasions of independent HTG from the bacterial ALKBHs was also supported. Therefore, all the findings in the present study will facilitate understanding of the evolution of RNA N6mA modifications and their important contributions to biological functions and diversity, with contributions from more experimental evidence in the future, especially on the HGT and gene duplication validification. However, one of the most commonly used and most sensitive analysis methods was used to analyze the public genome data of different species deposited in two databases in this study, and the genome with different quality may have an impact on the analysis. In the future, quality sequencing of the genomes of different species for systematic analysis and more analytical methods are recommended to validate our hypothesis.

## Data Availability

All raw data related to this study have been deposited in the Supplementary Materials.

## Supplementary Materials

The following supporting information can be downloaded at: https://www.mdpi.com/article/10.3390/biology11020214/s1, Figure S1: Phylogenetic tree of N6mA MTases. Figure S2: Phylogenetic tree of PCIF1. Figure S3: Phylogenetic tree of TRMO. Figure S4: Phylogenetic tree of WTAP. Figure S5: Phylogenetic tree of VRIMA. Figure S6: Phylogenetic tree of RBM15. Figure S7: Phylogenetic tree of HAKAI. Figure S8: Phylogenetic tree of ZC3H13. Figure S9: Phylogenetic tree of ALKBH family. Figure S10: Phylogenetic tree of YTH family. Figure S11: Phylogenetic tree of HNRNPs. Figure S12: Phylogenetic tree of IGF2BPs. Figure S13: Phylogenetic tree of FMRP1. Figure S14: Phylogenetic tree of ELAV1. Figure S15: Distribution of ALKB family in prokaryotes and eukaryotes. Figure S16: Structure based alignments of YTH domain containing proteins (h = alpha-helix, e = beta-sheet,) Consensus amino acid symbols are: conserved amino acids are in bold and uppercase letters; aliphatic (I, V, L): l; aromatic (Y, H, W, F): @; hydrophobic (W, F, Y, M, L, I, V, A, C, T, H): h; alcohol (S, T): o; polar residues (D, E, H, K, N, Q, R, S, T): p; tiny (A, G, C, S): t; small (A, G, C, S, V, N, D, T, P): s; bulky residues (E, F, I, K, L, M, Q, R, W, Y): b; positively charged (K, R, H): +; negatively charged (D, E): −; charged (D, E, K, R, H): c.). Table S1: N6mA modification writers, readers and erasers protein families as initial queries. Table S2: Classification of taxa. Table S3: Domains used to filter the homologues of N6mA modification writers, readers and erasers’ protein families. Table S4: Alignments criteria and Best fit model. Table S5: The m^6^A associated protein of prokaryotes in Ensemble database. Table S6: RMSD of N6mA MTases and ALKBH family. Supplementary File S1: Alignments for building phylogenetic trees.
