# Supplementary figures and images for: Evolutionary History of RNA Modifications at N6-Adenosine Originating from the R-M System in Eukaryotes and Prokaryotes
Source: Biology (Basel). 2022 Jan 28;11(2):214. doi: 10.3390/biology11020214 (PMC8868631; doi:10.3390/biology11020214)

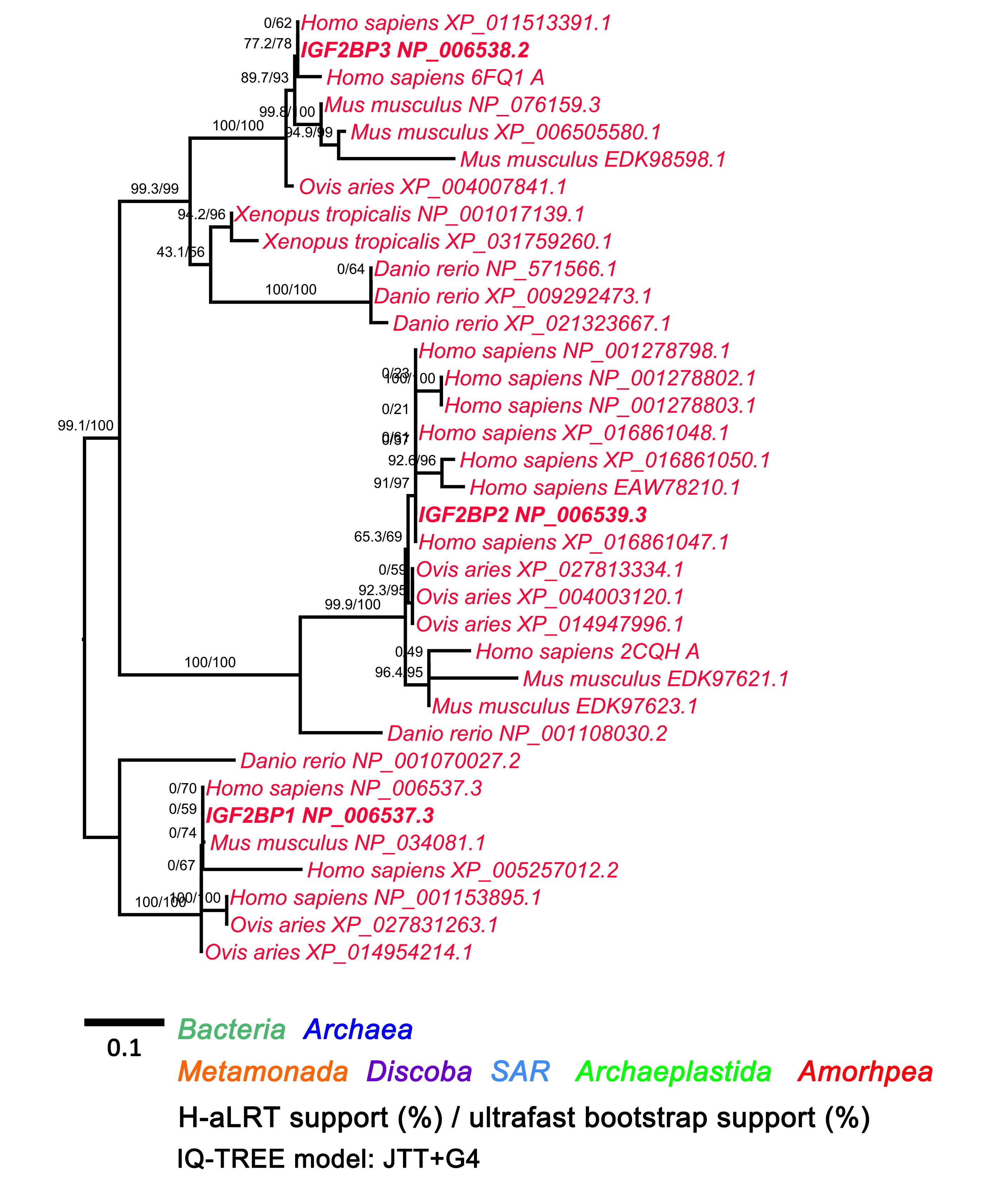

Supplement: Supplementary file 1 [file biology-11-00214-s001.zip › Figure S12.jpg]

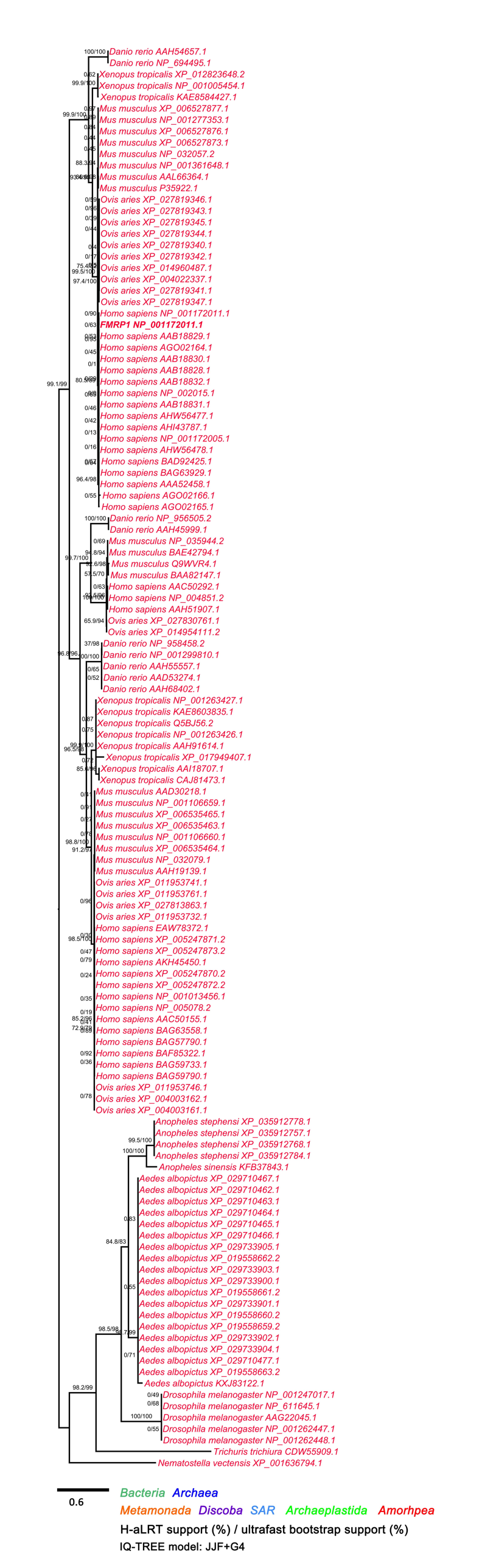

Supplement: Supplementary file 1 [file biology-11-00214-s001.zip › Figure S13.jpg]

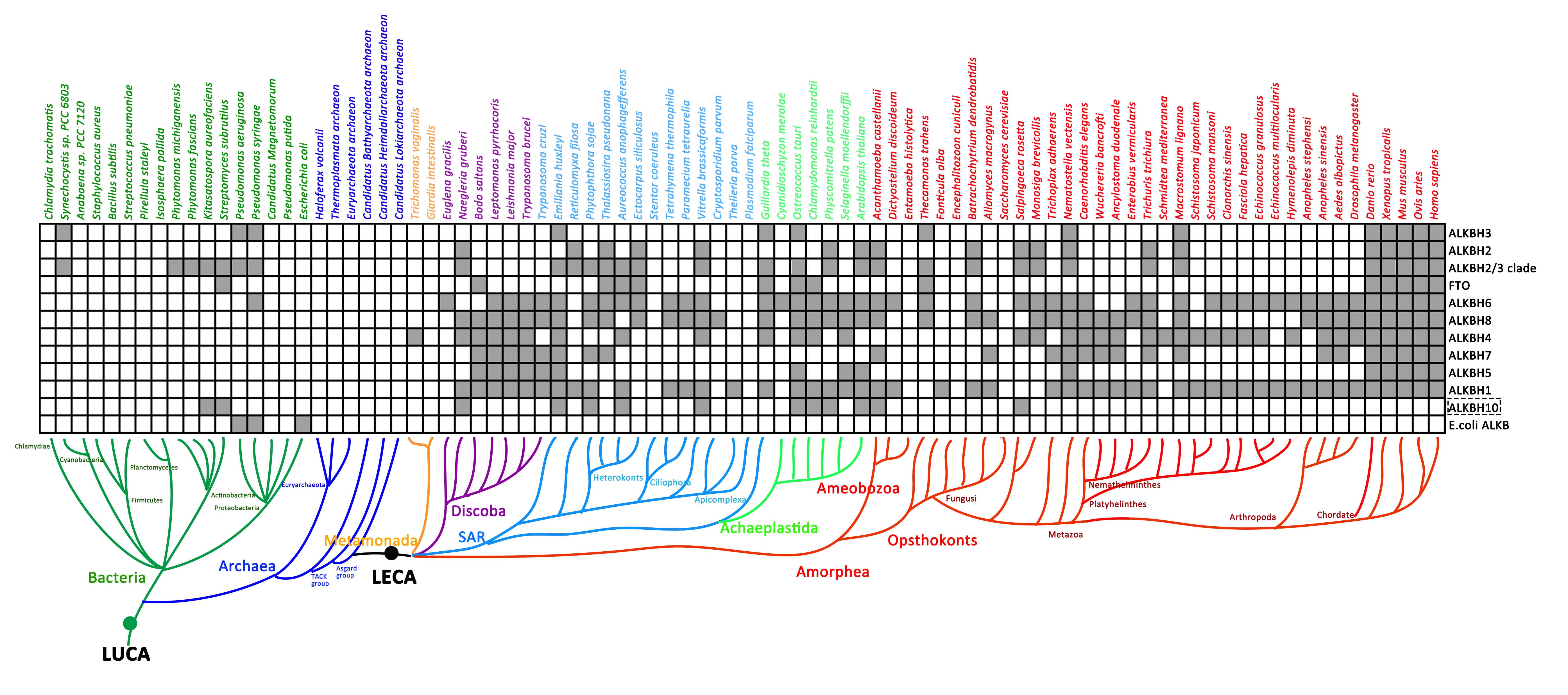

Supplement: Supplementary file 1 [file biology-11-00214-s001.zip › Figure S15.jpg]

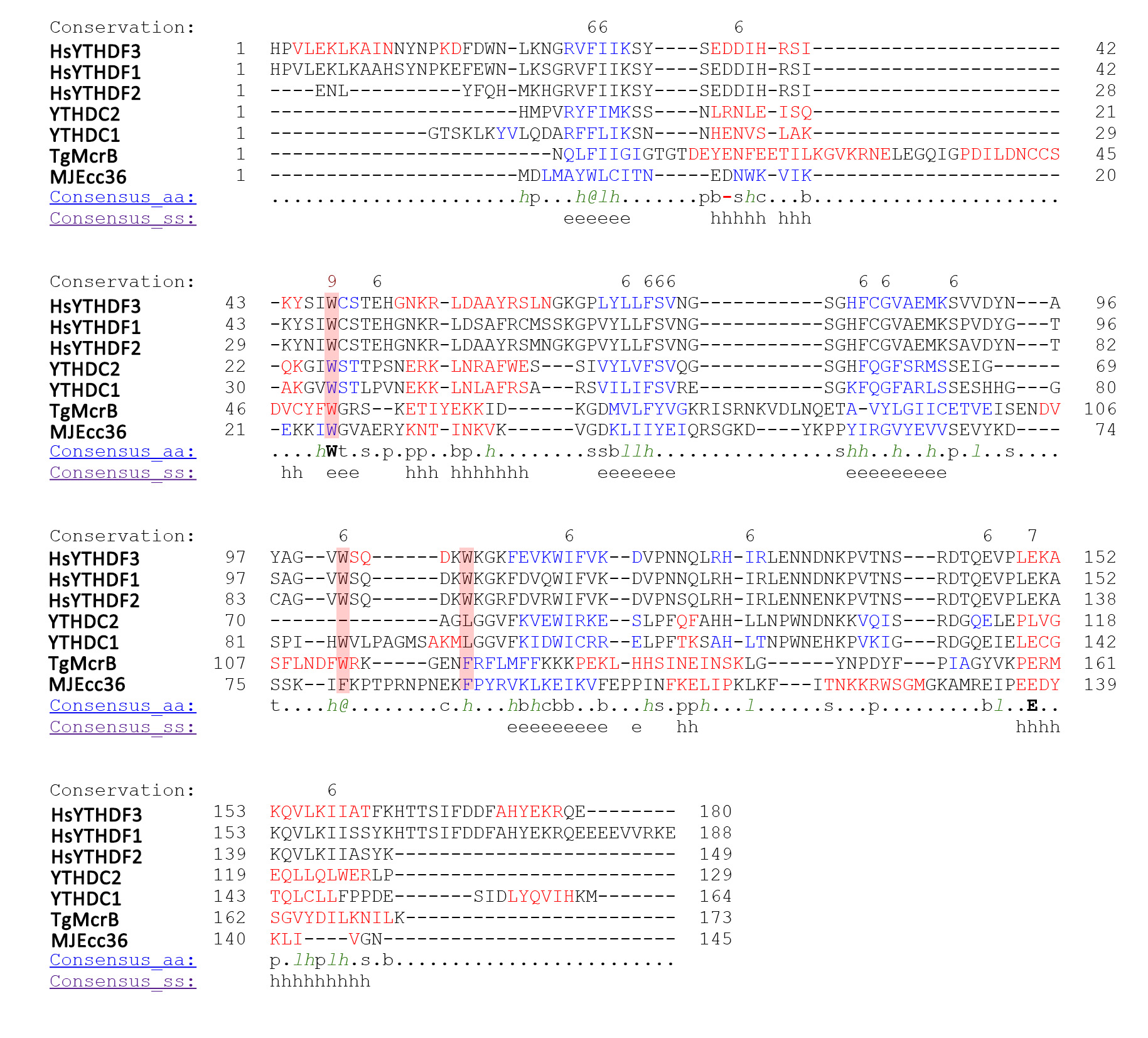

Supplement: Supplementary file 1 [file biology-11-00214-s001.zip › Figure S16.jpg]

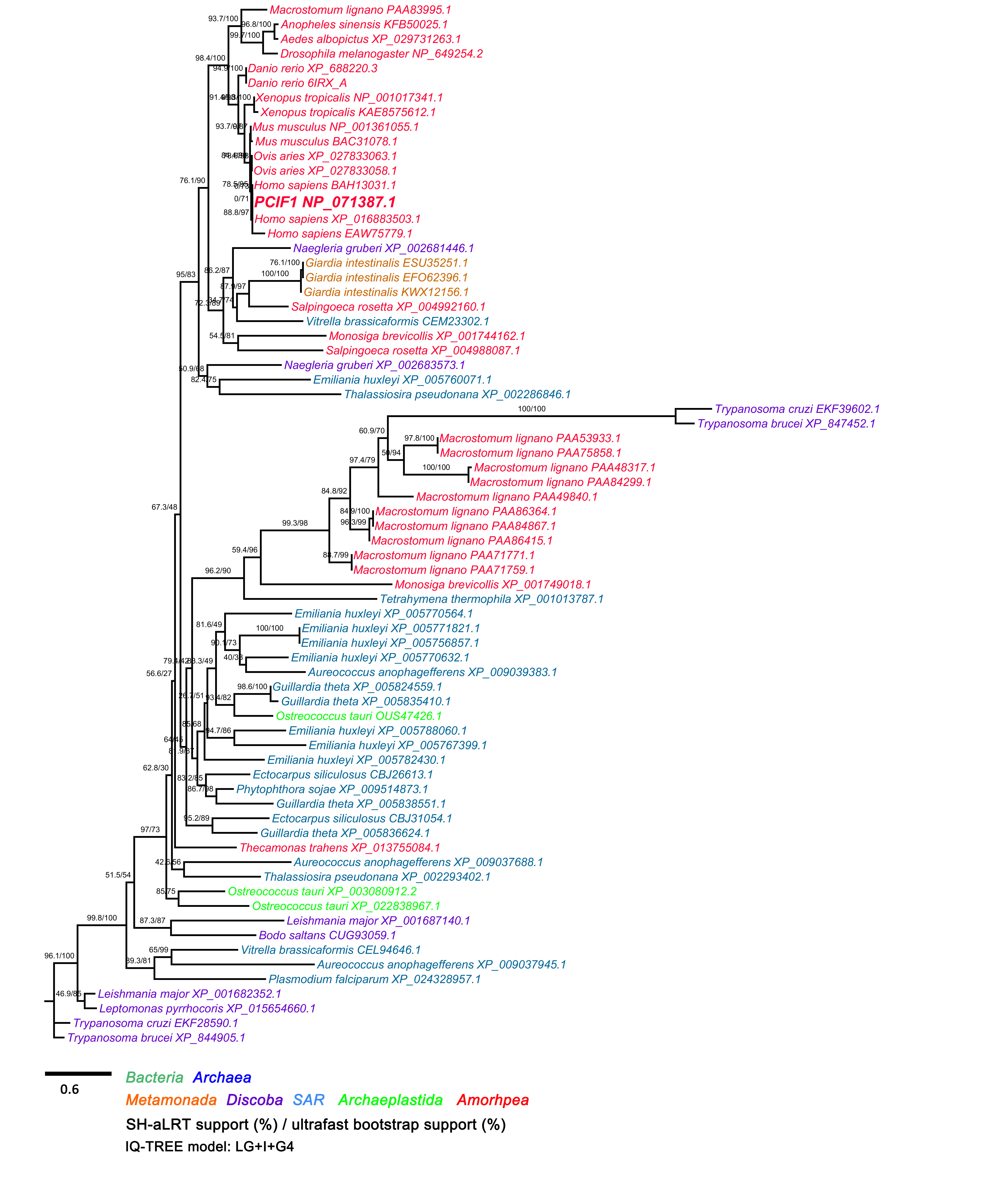

Supplement: Supplementary file 1 [file biology-11-00214-s001.zip › Figure S2.jpg]

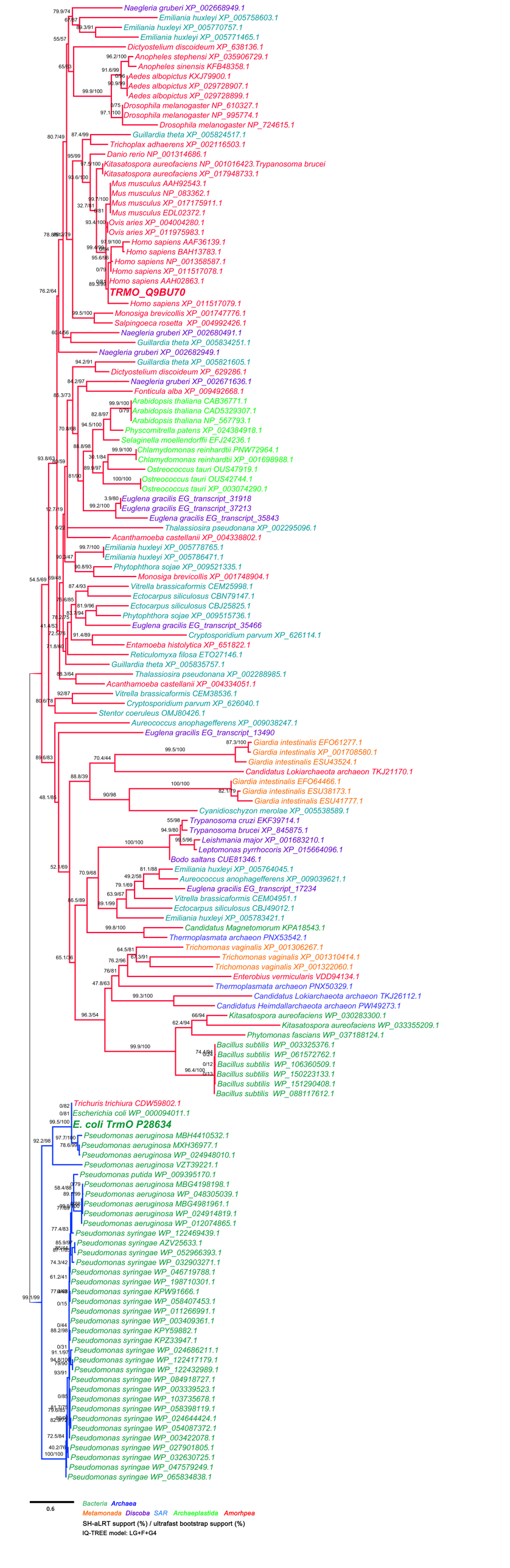

Supplement: Supplementary file 1 [file biology-11-00214-s001.zip › Figure S3.jpg]

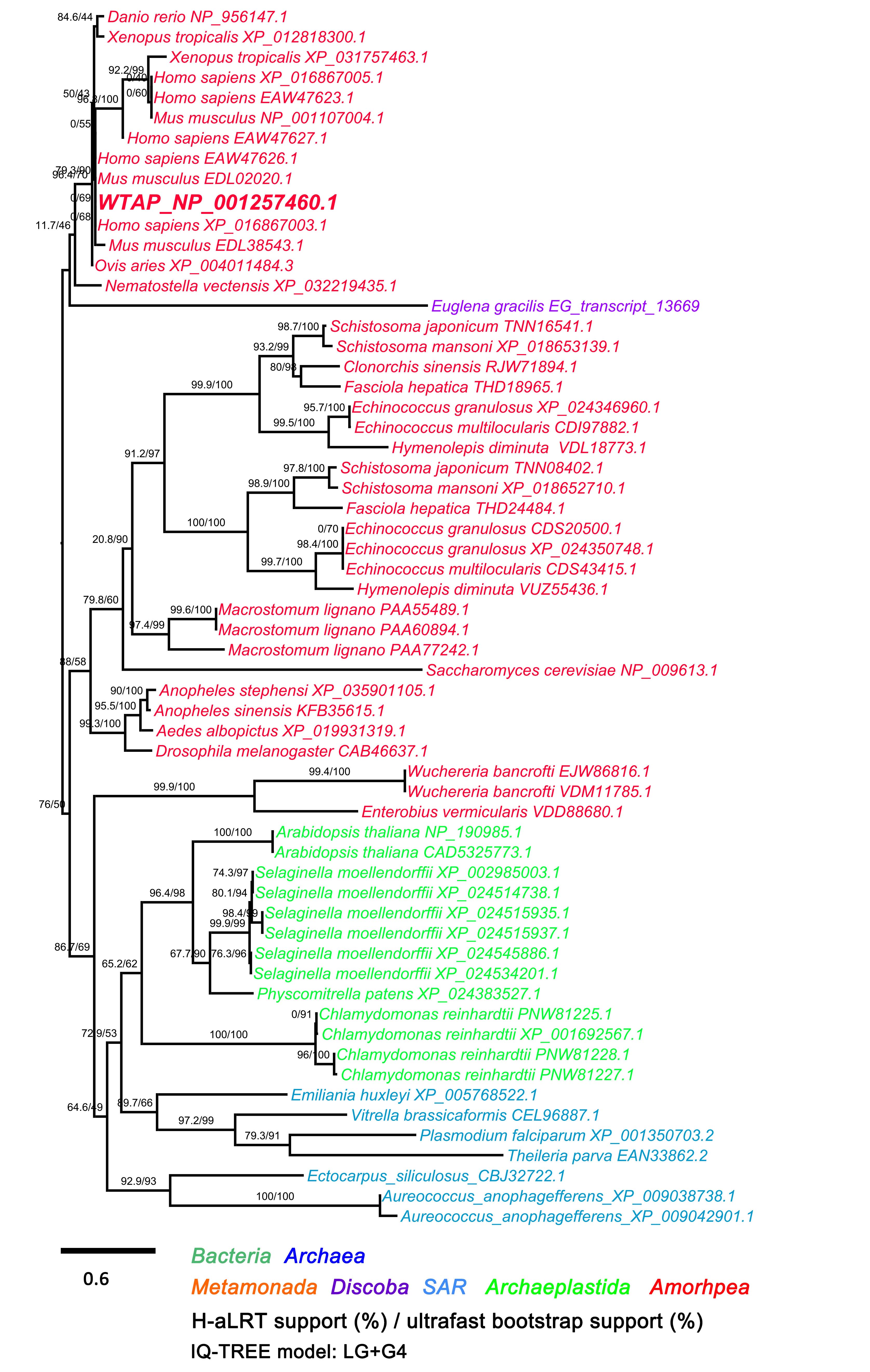

Supplement: Supplementary file 1 [file biology-11-00214-s001.zip › Figure S4.jpg]

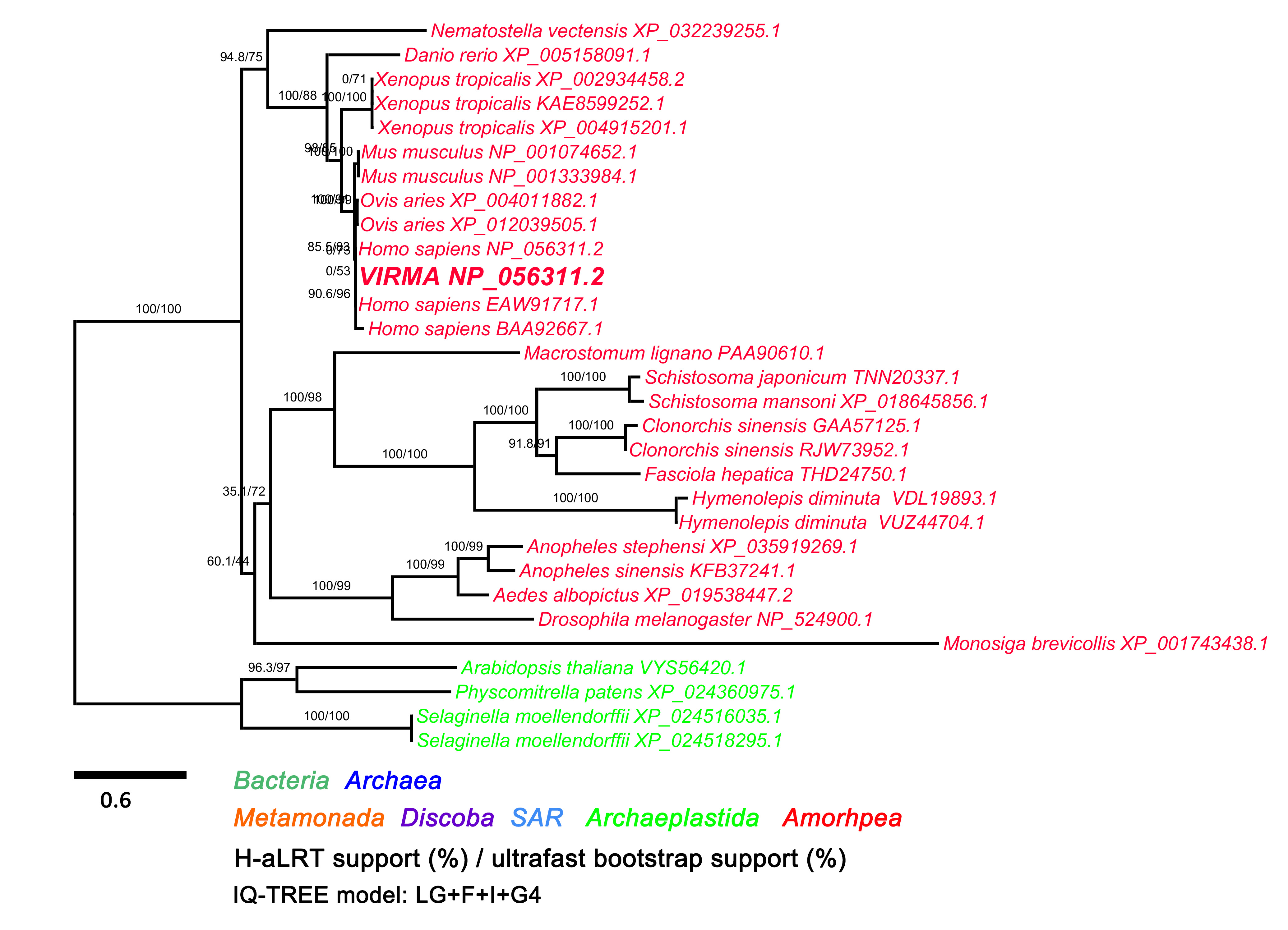

Supplement: Supplementary file 1 [file biology-11-00214-s001.zip › Figure S5.jpg]

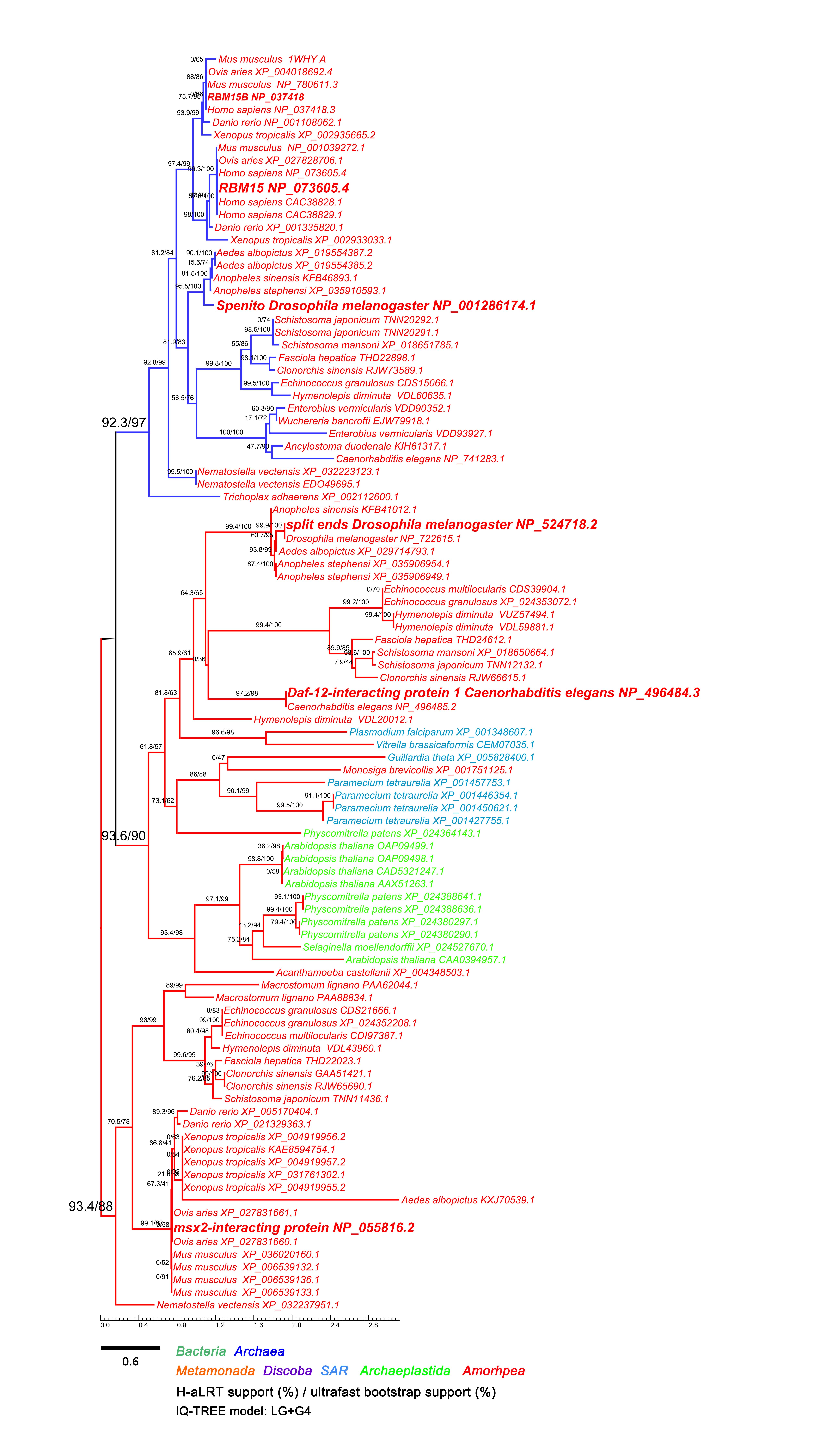

Supplement: Supplementary file 1 [file biology-11-00214-s001.zip › Figure S6.jpg]

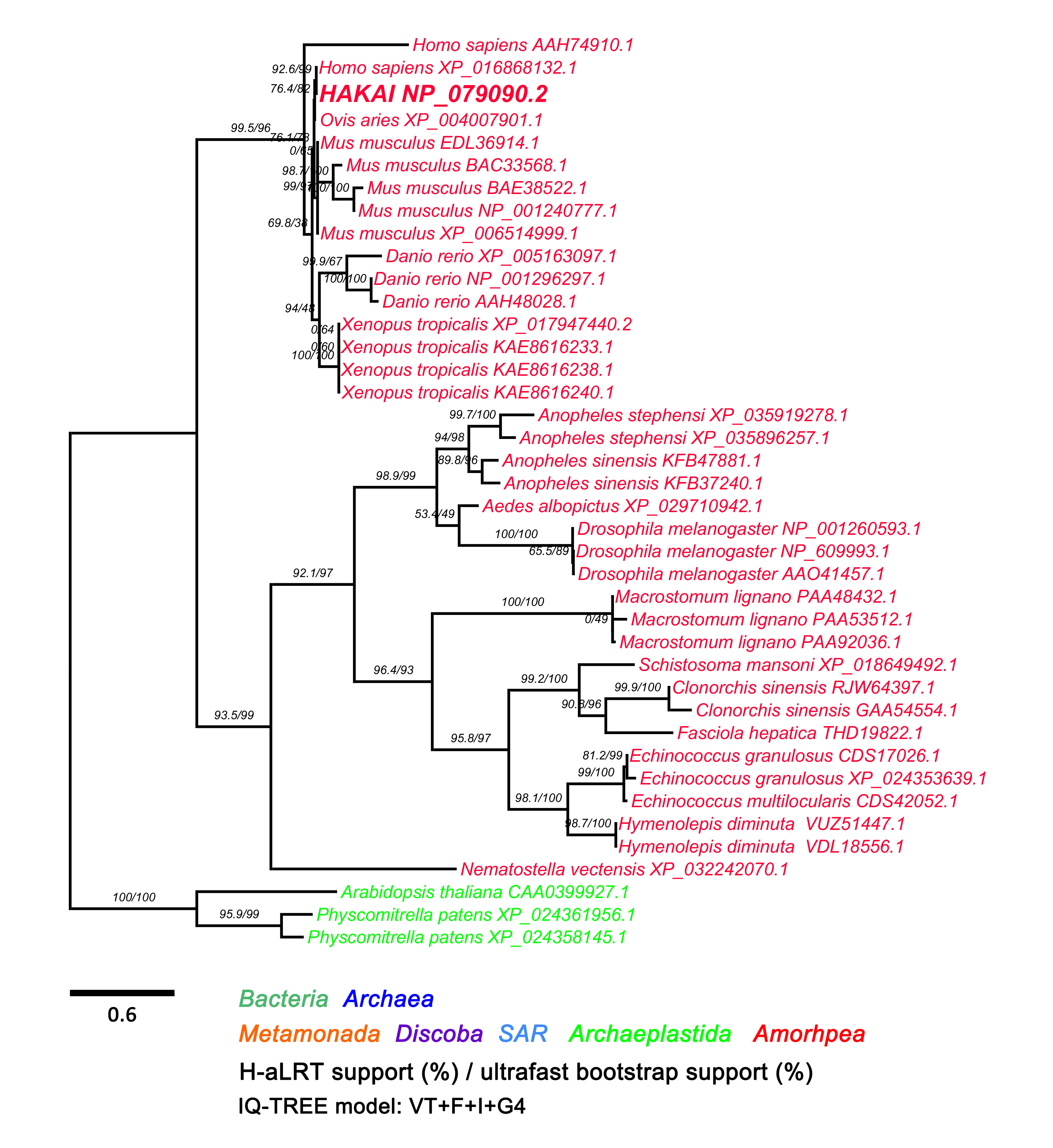

Supplement: Supplementary file 1 [file biology-11-00214-s001.zip › Figure S7.jpg]

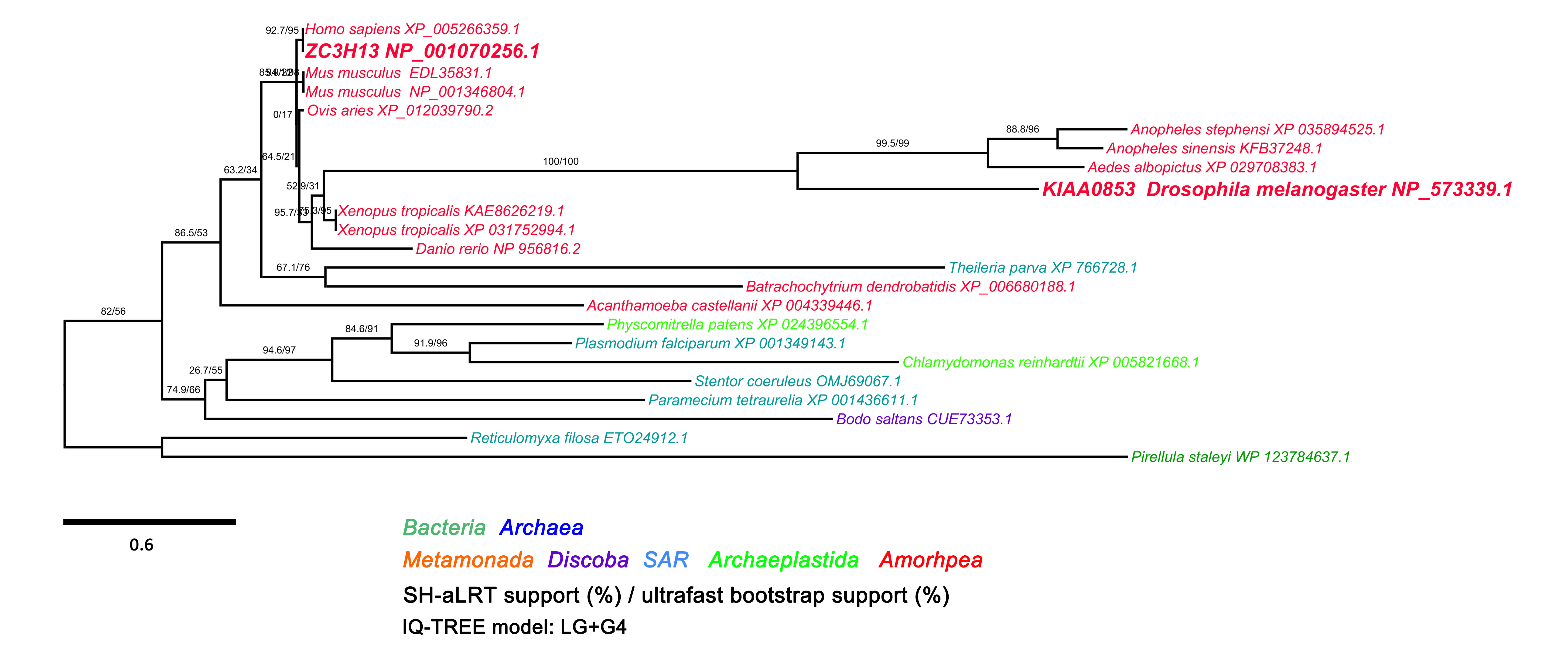

Supplement: Supplementary file 1 [file biology-11-00214-s001.zip › Figure S8.jpg]
